# Hypofractionated versus standard chemoradiotherapy in the definitive treatment of uterine cervix cancer: interim results of a randomized controlled clinical trial

**DOI:** 10.1007/s00432-023-05563-8

**Published:** 2024-01-20

**Authors:** Afsane Maddah Safaei, Ebrahim Esmati, Marzieh Gomar, Setareh Akhavan, Shahrzad Sheikh Hasani, Mona Malekzadeh Moghani, Narges Zamani, Maryam Moshtaghi, Mahrooz Malek, Fatemeh Jafari, Azadeh Sharifian, Kasra Kolahdouzan

**Affiliations:** 1 Radiation Oncology Research Center (RORC), Cancer Research Institute, Tehran University of Medical Sciences, Tehran, Iran; 2 Department of Obstetrics and Gynecology, Vali-Asr Reproductive Health Research Center, Tehran University of Medical Sciences, Tehran, Iran; 3 Department of Oncologic Gynecology, Vali-Asr Hospital, Tehran University of Medical Sciences, Tehran, Iran; 4 Department of Radiation Oncology, Faculty of Medicine, Shahid Beheshti University of Medical Sciences, Tehran, Iran; 5 Radiation Oncology Department, Booali Hospital, Tehran, Iran; 6 Advanced Diagnostic and Interventional Radiology Research Center (ADIR), Radiology Department, Imam Khomeini Hospital Complex (IKHC), Tehran University of Medical Sciences, Tehran, Iran

**Keywords:** Cervix cancer, Chemoradiotherapy, Brachytherapy, Hypofractionation

## Abstract

**Purpose:**

Concurrent chemoradiation has been the mainstay of treatment for cervix cancer. We aimed to evaluate the non-inferiority of hypofractionated chemoradiation.

**Methods:**

This study was designed as a phase 2, 1:1 randomized, investigator-blinded, controlled, non-inferiority trial and we report the interim results after 50% accrual. Cervical cancer patients with FIGO stages IIA–IIIC were recruited from April 2021 to September 2022. The intervention consisted of 40 Gy of 3D-conformal radiation therapy (RT) in 15 fractions over 3 weeks. In the control group, patients received standard chemoradiation of 45 Gy in 25 fractions over 5 weeks. Both groups received concurrent weekly cisplatin (40 mg/m^2^). Intravaginal brachytherapy of 28 Gy in 4 weekly fractions was delivered starting 1 week after the end of chemoradiation. The primary outcome was complete clinical response(CCR) at 3 months. Secondary outcomes included acute gastrointestinal (GI), genitourinary(GU), skin, and hematologic toxicities. A *p* value less than 0.05 was considered significant for analyses.

**Results:**

59 patients were randomized; 30 in the control group and 29 in the intervention group. 20/30 (66.7%) of the patients in the control group and 19/29 (65.5%) in the intervention group achieved a CCR (absolute difference of 0.011, 95% CI − 0.23 to 0.25, *p* value: 0.13). There was a significantly higher rate of acute grade ≥ 3 GI toxicity in the intervention group (27.6%) compared with the control group (6.7%) (*p* value 0.032).

**Conclusions:**

Despite an absolute difference of 1.1% in the 3-month CCR, our interim analysis failed to show the non-inferiority of the hypofractionated chemoradiation. Due to the higher GI toxicities, we will continue this trial using intensity-modulated radiation therapy.

**Registration number and date:**

ClinicalTrials.gov: NCT04831437, 2021.4.1.

## Introduction

Uterine cervix cancer is the fourth most common malignancy and cause of death in women, and a major cause of disease burden especially in the developing countries (International Agency for Research on Cancer (IARC) 2020). Unfortunately, this higher prevalence in the lower income countries is attributed to a lack of systematic human papillomavirus (HPV) vaccination programs and lower access of sexually active women in these countries to prevention and screening services (Cohen et al. 2019). Concurrent chemoradiation followed by intravaginal brachytherapy has been the mainstay of treatment for locally advanced disease with surgery reserved for very early stage disease or refractory/recurrent disease. Hypofractionated radiotherapy has been used and become the standard of care in different cancer sites like prostate, rectum, breast, melanoma, GBM, etc., and offers radiobiologic superiority for tissues with a low *α*/*β* ratio. It also causes a higher dose per fraction to be delivered in a shorter overall treatment time (OTT) likely mitigating the effects of repopulation of malignant cells. From a healthcare economics point of view, shorter OTT translates into a lower workforce required, decreased costs, and more convenience for the patients.

As the COVID-19 pandemic broke out in late 2019, many patients were concerned about the long treatment duration required in radiotherapy facilities, and many physicians turned toward hypofractionated radiotherapy schedules wherever possible (Piras et al. 2022).

There is not any randomized trial for hypofractionation in uterine cervix cancer and only a few small retrospective studies reporting single-center experiences can be found in the literature (Campbell et al. 2000; Huilgol et al. 1988; Muckaden et al. 2002; Viegas et al. 2004). These studies have all reported similar control rates between hypofractionated and conventional chemoradiation regimens. There are also very few retrospective studies reporting successful administration of hypofractionated regimens for the palliative treatment of advanced cervical disease in the elderly (Kiattikul et al. 2022).

Incited by the COVID-19 era, we designed the current randomized clinical trial to evaluate whether hypofractionated chemoradiation is truly non-inferior to the conventional treatment protocols.

## Materials and methods

### Trial design

This study was a phase 2, 1:1 randomized, investigator-blinded, controlled, parallel-group non-inferiority trial of hypofractionation in definitive concurrent chemoradiation of patients with uterine cervix cancer, conducted in a single center in Iran. The study protocol was reviewed and approved by the institutional review board and ethics committee (IR.TUMS.IKHC.REC.1399.514).

### Participants

All female patients with a biopsy-proven diagnosis of cervical cancer were included if: (1) provided written informed consent to enter the study, (2) aged between 18 and 85, (3) pathologically diagnosed with squamous cell carcinoma, adenocarcinoma, or adenosquamous carcinoma of the uterine cervix, (4) confirmed with magnetic resonance imaging (MRI) to have International Federation of Gynecology and Obstetrics (FIGO) 2018 stages of IIA to IIIC1, (5) were medically eligible to receive cisplatin, and (6) had Eastern Cooperative Oncology Group (ECOG) performance status of 0–2.

Patients were excluded in case of a known history of inflammatory bowel disease and connective tissue disorders. In addition, those with a previous history of pelvic radiotherapy or hysterectomy were deemed ineligible to enter the study. We excluded patients with stage IIIB and hydronephrosis if they had a creatinine clearance rate of < 30 ml/min. In addition, stage IIIC1 patients with more than three MRI-determined lymphadenopathies and/or with at least one lymph node with a short-axis diameter of 3 cm and/or lymphadenopathy located in the common iliac chains were excluded.

The study was conducted in a tertiary academic healthcare facility (radiation oncology department of Imam Khomeini Hospital Complex in Tehran, Iran), from April 2021 to September 2022 (end of recruitment for interim analysis).

### Study arms

The intervention consisted of 3D conformal radiation therapy (RT) to a total dose of 40 grays (Gy) in 15 fractions over 3 weeks (5 fractions per week) concurrently with 40 mg per square meter of body surface area of cisplatin infused intravenously once a week for a total of 3 infusions. Stage IIIC1 patients in the intervention group received an additional boost of 8 Gy in 3 fractions to the gross lymphadenopathies immediately after the conclusion of RT. In the control group, patients received standard chemoradiation of 45 Gy in 25 fractions over 5 weeks concurrently with a maximum of 5 weekly infusions of 40 mg/m^2^ cisplatin. In addition, stage IIIC1 patients in the control group received an additional boost of 9 Gy in 5 fractions to the gross lymphadenopathies after RT. All patients were required to undergo intravaginal radiotherapy (IVRT) starting 1 week from the end of RT to a total dose of 28 Gy in 4 weekly fractions.

After randomization of participants, simulation abdominopelvic computed tomography (CT) scan with a slice thickness of 5 mm was acquired from all patients in the supine position with a full bladder and an empty rectum. Images were transported to the treatment planning system. Contouring of gross, clinical, and planning target volumes (GTV, CTV, and PTV, respectively) was done based on the NRG-GY006 protocol (Institute and Oncology 2016). Briefly, this included contouring the GTV + uterus + cervix as CTV1, parametria + upper half of vagina as CTV2, and common iliac, external iliac, internal iliac, obturator, and presacral nodes as CTV3. Finally, a volumetric margin of 15 mm, 10 mm, and 5 mm was given to form the respective PTVs. In the case of malignant regional lymphadenopathy, MR images were fused with the simulation CT scan to aid in contouring of gross nodal involvement and 1 cm anatomic margin was given to create a boost CTV with a final 5 mm volumetric margin to form the boost PTV. A final PTV dose coverage within 95–107% of the prescribed dose was deemed acceptable. Special care was given to not exceed the organ at risk dose limits, especially in patients with stage IIIC1 receiving a boost dose. These dose constraints included maximum dose ≤ 50 Gy and D30% ≤ 40 Gy for the bowel space, D50% ≤ 45 Gy, D60% ≤ 30 Gy, maximum dose ≤ 50 Gy for the rectum, and D50% ≤ 45 Gy, maximum dose ≤ 50 Gy for the bladder. Treatment was delivered using the Elekta Synergy^®^ radiotherapy device with 18 MV photons to the planning target volume with a 4-field box technique shielding the organs at risk with the multileaf collimator. Daily electronic portal imaging was acquired to verify the patient setup accuracy.

One week after completion of RT, patients were required to undergo a second MRI to evaluate tumor shrinkage for brachytherapy delivery. Tandem and ovoids were placed in the operating room with the patient under general anesthesia or deep sedation. In case of an initially bulky tumor or gross residue after RT, additional catheters were inserted interstitially to help the coverage of the high-risk CTV (HR-CTV). Simulation CT scans were acquired and fused with MRI for contouring and planning based on GEC–ESTRO guidelines (Haie-Meder et al. 2005). A final dose of 28 Gy in 4 fractions was anticipated to adequately cover the high-risk CTV.

### Outcomes

The primary outcome was complete clinical response (CCR) at 3 months after the last IVRT delivery session, defined as no evidence of residual tumor in gadolinium-enhanced pelvic MRI with vaginal gel infusion reported by an experienced radiologist with more than 10 years of experience in gynecologic malignancies. Secondary outcomes included acute gastrointestinal (GI), genitourinary (GU), skin, and hematologic toxicities within 3 months as defined by the Common Terminology Criteria for Adverse Events (CTCAE) Version 5.0 assessed objectively by the study investigators during treatment and within 3 months after treatment.

### Sample size calculation

Based on an institutional data analysis on over 180 cervical cancer patients, a complete response rate over 3 months was predicted for 65% of the similar stage (IIA–IIIC) patients receiving definitive chemoradiation (Tabatabaei et al. 2022). Due to a shorter expected overall treatment time (OTT) (7 weeks versus 9 weeks) and higher predicted patient compliance, we expected a 10% higher complete response rate in the experimental group (75%). Considering a margin of 15% acceptable for determining the non-inferiority of the experimental group with a 90% power (with a one-sided type-I error of 5%), a sample size of 57 patients was required in each group. We initially planned to conduct an interim analysis after 50% of the participants were accrued and stopping rules were based on the O'Brien–Fleming boundary of *α* = 0.005 (O'Brien and Fleming 1979).

### Randomization and allocation

A web-based computer program (Sealed Envelope Ltd 2022) was used for the generation of a randomization list with permutated blocks method with FIGO stage being used as the stratification factor for block sizes of 6. Patients were allocated based on the number sequence in the randomization block. Only one investigator had access to the randomization list and allocation information was concealed from the investigators in charge of RT treatment planning verification. The physician in charge of IVRT treatment planning was not blinded to the treatment groups. Neither the patients, nor the treating physicians were blinded due to the nature of treatment, but the radiologist assigned for response evaluation and the data analyzing investigator were blinded to the study arms.

### Statistical analysis

Basic descriptive tests and frequencies were acquired for the qualitative data. Normality was tested for the quantitative data using the Kolmogorov–Smirnov test. Pearson's Chi-squared test was used to test the correlation between two categorical parameters. Independent samples *t* test and Mann–Whitney *U* tests were used for comparison of quantitative parameters in the two study groups for normally and non-normally distributed parameters, respectively.

Efficacy and safety analyses were performed in the intention-to-treat population. Using the Miettinen and Nurminen method, the two-sided 95% confidence interval for the absolute difference of complete response rate was calculated. The test statistic (*Z*_cu_) was calculated based on the Kawasaki method for non-inferiority margin *p* value (Kawasaki et al. 2010).

The subgroup analyses were done in the per-protocol population. We formed a per-protocol population after excluding patients who did not receive all of the assigned external RT schedules, received the opposite treatment (due to toxicity or patient withdrawal), or were lost to the follow-up. Odds ratios and their 95% confidence intervals in different subgroups were calculated using Mantel–Haenszel estimates and an interaction *p* value was reported using the binomial regression analysis.

All analyses were done using IBM SPSS statistics version 26, and R version 4.2.2. Study plots were created using GraphPad Prism version 8.0.

## Results

**Fig. 1:**
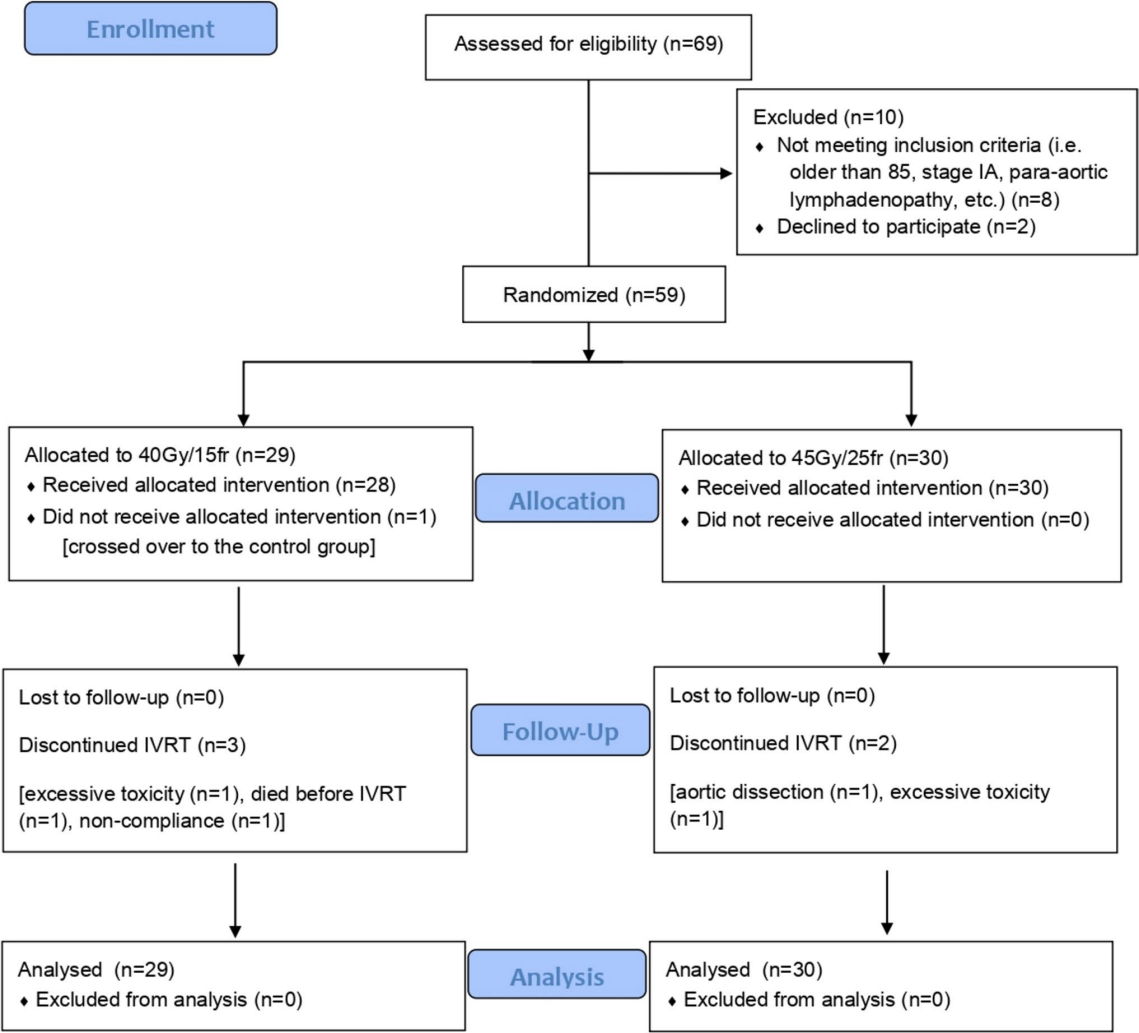
CONSORT flow diagram. *IVRT*, intravaginal brachytherapy

Sixty-nine patients were initially assessed for inclusion in the study. Ten patients were excluded due to a violation of the study eligibility criteria. Finally, 59 patients were randomized; 30 in the control group and 29 in the intervention group (the intention-to-treat population) (Fig. 1).

One patient changed treatment arm (from intervention to the control group) after three fractions of RT. Five patients did not receive IVRT (2 control, 3 intervention); one because of aortic dissection, two due to grade 5 GI toxicity, one due to grade 4 GI toxicity (did not finish RT either), and one due to non-compliance. In addition, one control patient died before response evaluation, and another control patient had uterine rupture (due to malignant residual disease) before response evaluation.

Eventually, 27 control and 26 intervention patients received the intended chemoradiation and reached the 3-month evaluation, which translates into 25 interventions and 27 controls when excluding the patient that changed groups from the intervention to the control group (the per-protocol population).

### Baseline clinical characteristics

The mean age of study participants was 53 (SD = 14.4). The most common presenting symptom was vaginal bleeding in 51 patients (86.4%), followed by post-coital bleeding/pain in 7 (11.9%), and back pain in 1 (1.7%) patient. 89.8% (53 patients) were diagnosed with SCC, 8.5% (5 patients) with adenocarcinoma, and 1.7% (1 patient) with adenosquamous carcinoma. 81.3% of the diagnoses were HPV associated (p16 positive). The time interval between the first diagnosis (defined as the biopsy date) to the RT start date was 63.5 ± 33.3 days in the intervention group and 58.5 ± 23.6 days in the control group (*p* value: 0.51). In addition, the overall treatment time from the RT start date to the end of IVRT was significantly shorter in the intervention group (59.6 ± 14.5 days) versus the control group (74.8 ± 10.9 days), *p* value: 0.0001.

The most common FIGO 2018 clinical stage of patients was IIIC (54%), followed by IIB (32%), and IIA (8%). Patients were not statistically different in the two groups regarding baseline characteristics, comorbidities, or lab parameters (Table 1). MRI-based tumor volume was calculated as a median of 31 cc [interquartile range 14–50 cc] for the control group and 29 cc [16–49 cc] for the intervention group (*p* = 0.96). Maximum tumor dimension (Dmax) was 4.7 ± 1.4 cm for the control and 4.7 ± 1.3 cm for the intervention group (*p* = 0.87).

**Table 1:** Baseline demographic and clinical characteristics of the study participants

	All (*n* = 59)	Intervention (*n* = 29)	Control (*n* = 30)	*p* value
Age	53 ± 14.4 [mi*n* = 28, max = 85]	55.1 ± 13.9	51.1 ± 14.8	0.29
Pathology				0.55
SCC	53 (89.8%)	26 (89.7%)	27 (90%)	
ADC	5 (8.5%)	2 (6.9%)	3 (10%)	
Adenosquamous	1 (1.7%)	1 (3.4%)	0	
Stage				0.9
IIA	5 (8.5%)	2 (6.9%)	3 (10%)	
IIB	19 (32.2%)	9 (31%)	10 (33.3%)	
IIIA	0	0	0	
IIIB	3 (5.1%)	2 (6.9%)	1 (3.3%)	
IIIC	32 (54.2%)	16 (55.2%)	16 (53.3%)	
BMI (kg/m^2^)	27.8 ± 5.6	27.4 ± 5.3	28.2 ± 5.9	0.59
Lymph nodes				
#	32 (54.2%)	16 (55.2%)	16 (53.3%)	0.47
1 LN	11 (18.6%)	4 (13.8%)	7 (23.3%)	
2 LNs	13 (22%)	8 (27.6%)	5 (16.7%)	
3 LNs	8 (13.6%)	4 (13.8%)	4 (13.3%)	
Laterality				0.72
Unilateral	17 (28.8%)	8 (27.6%)	9 (30%)	
Bilateral	15 (25.4%)	8 (27.6%)	7 (23.3%)	
SAD				0.52
≤ 1 cm	22 (37.3%)	12 (41.4%)	10 (33.3%)	
> 1 cm, ≤ 2 cm	9 (15.2%)	4 (13.8%)	5 (16.7%)	
> 2 cm, < 3 cm	1 (1.7%)	0	1 (3.3%)	
Comorbidity				
DM	15 (25.4%)	9 (31%)	6 (20%)	0.33
HTN	19 (32.2%)	10 (34.5%)	9 (30%)	0.71
HLP	6 (10.2%)	2 (6.9%)	4 (13.3%)	0.41
IHD	8 (13.6%)	3 (10.3%)	5 (16.7%)	0.48
Other	2 (3.4%)	1 (3.4%)	1 (3.3%)	0.98
Lab				
WBC	8330 ± 4027	9046 ± 4791	7614 ± 3003	0.19
Hb (g/dL)	12.2 ± 1.7	12.3 ± 1.6	12 ± 1.7	0.43
Plt*10^3^	283 ± 104	303 ± 109	263 ± 97	0.16
Cr (mg/dL)	0.88 ± 0.25	0.85 ± 0.23	0.92 ± 0.27	0.29
GFR (ml/min)	89.4 ± 35.9	88.6 ± 37.5	90.3 ± 34.8	0.86

*ADC*, adenocarcinoma; *BMI*, body mass index; *Cr*, creatinine; *DM*, diabetes mellitus; *GFR*, glomerular filtration rate; *HLP*, hyperlipidemia; *HTN*, hypertension; *IHD*, ischemic heart disease; *LN*, lymph node; *Plt*, platelet count; *SAD*, short axis diameter; *SCC*, squamous cell carcinoma; *WBC*, white blood cell count

### Complete clinical response

**Fig. 2:**
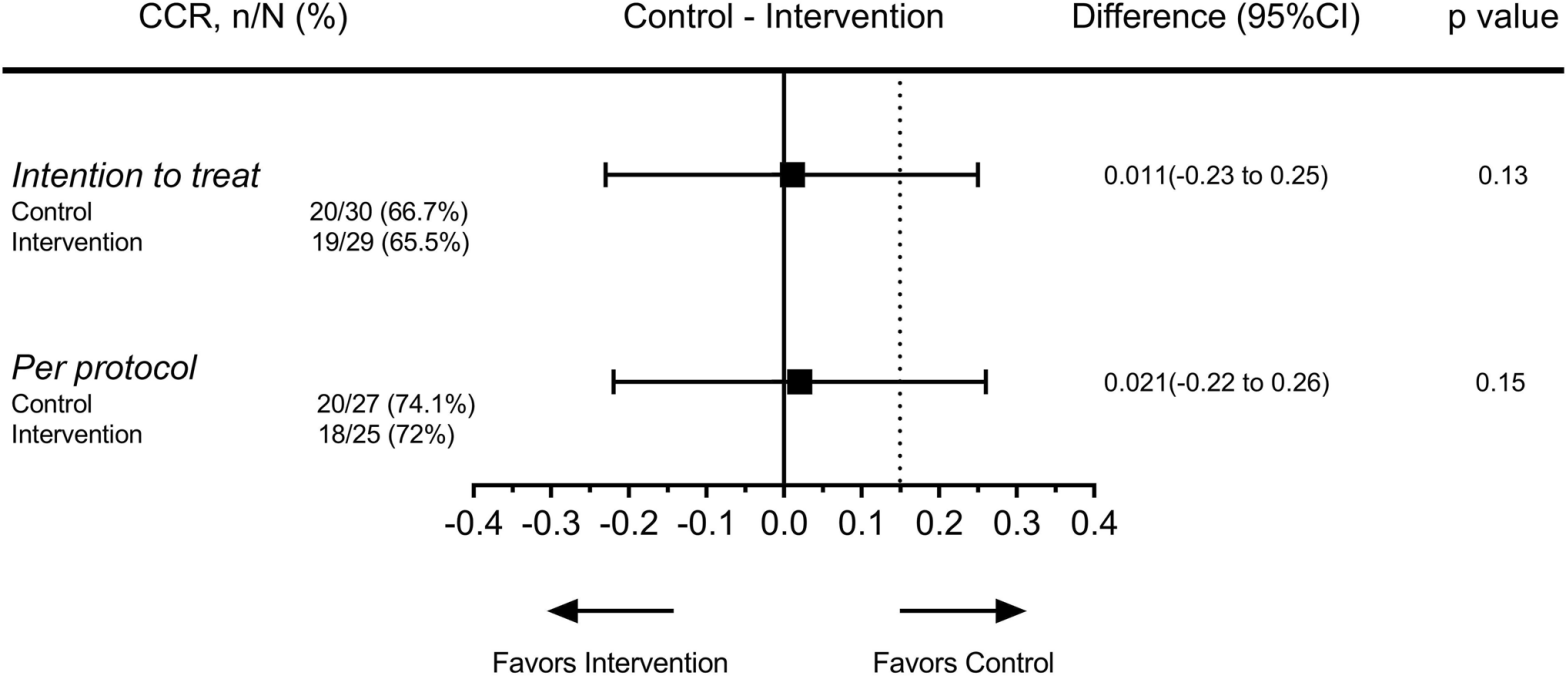
Effect of chemoradiation fractionation on complete clinical response. The absolute difference of complete clinical response rate between the two groups is shown by the filled squares and the extremities indicate the 95% confidence interval. The dotted line shows the predefined 15% margin of non-inferiority. Analysis is done in both intention-to-treat and per-protocol populations. *CCR*, complete clinical response

20/30 (66.7%) of the patients in the control group and 19/29 (65.5%) of the patients in the intervention group achieved a CCR at 3 months after IVRT (Absolute difference of 0.011, 95% CI − 0.23 to 0.25, *Z*_cu_ = 1.12, *p* value 0.13). In the per-protocol population, 20/27 (74.1%) of control, and 18/25 (72%) of intervention patients had a 3-month complete response, with an absolute difference of 0.02, 95% CI − 0.22 to 0.26, *Z*_cu_ = 1.04, *p* value 0.15 (Fig. 2).

**Fig. 3:**
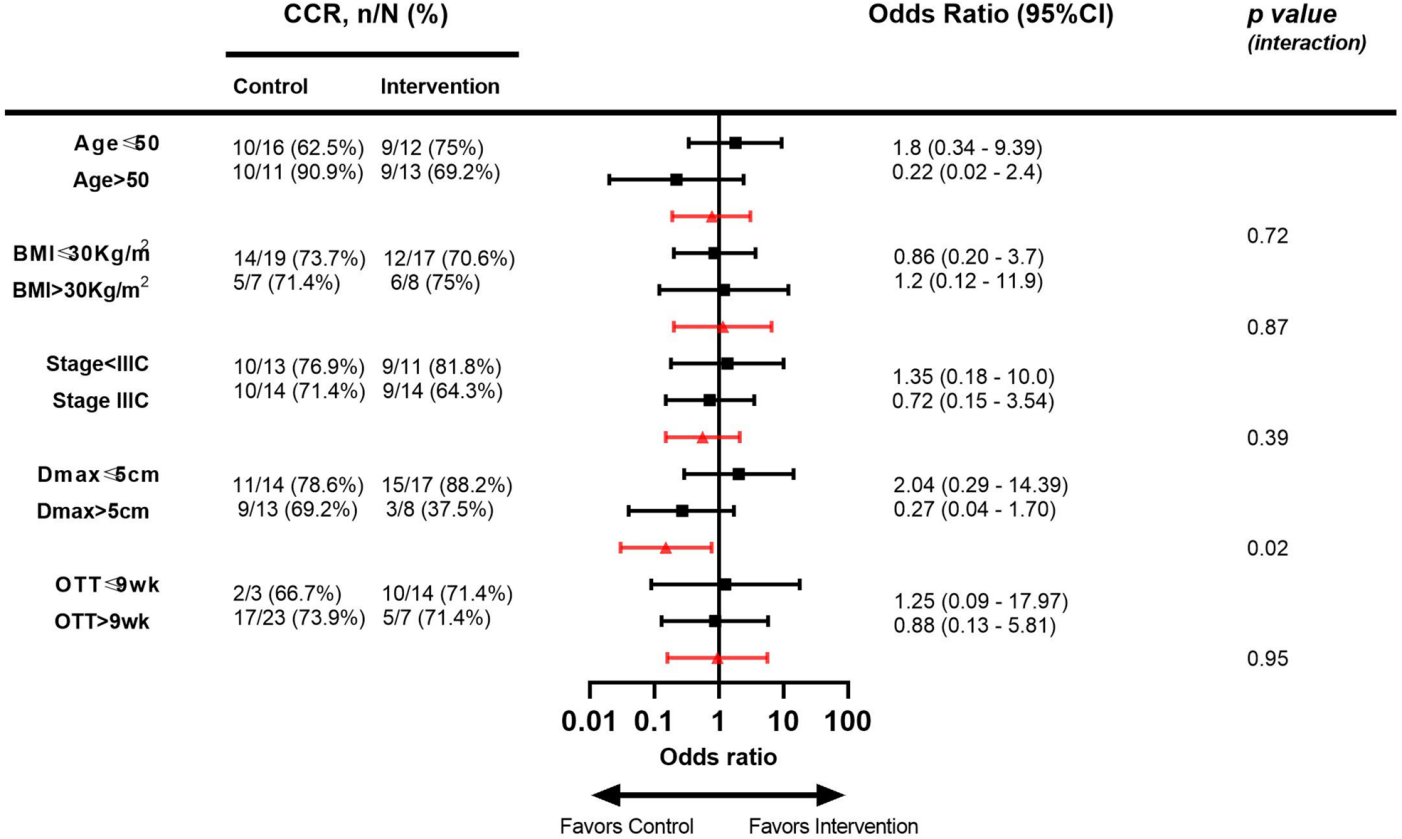
Effect of chemoradiation fractionation on complete clinical response by age, body mass index, FIGO stage, maximum tumor dimension, and overall treatment time. The filled squares indicate the odds ratios of achieving complete clinical response in each study arm within the specified subgroup of patients and the black lines indicate the 95% confidence intervals. The red triangles indicate the interaction odds ratios of achieving complete clinical response when receiving the intervention in the specified subgroup and the red lines indicate the 95% confidence intervals of the interaction odds ratios. *BMI*, body mass index; *Dmax*, maximum tumor dimension; *OTT*, overall treatment time

In an unplanned subgroup analysis in the per-protocol population stratifying the treatment groups according to age, stage, maximum tumor size, BMI, and OTT, patients with maximum tumor size larger than 5 cm achieved higher rates of CCR in the control group versus the intervention group in contrast to the superior results seen in patients with tumor size ≤ 5 cm receiving the intervention (interaction *p* value 0.02). There were no statistically significant findings in the other subgroups (Fig. 3).

### Treatment toxicity

Twelve control patients (40%) received less than 5 cycles of weekly cisplatin, and dose reduction was required in 9/30 (30%) during treatment, whereas 6/29 intervention patients (20.7%) received less than 3 cycles of cisplatin (*p* value 0.11), and dose reduction was required in 7/29 (24.1%) of the patients. A 50% cisplatin dose reduction was applied for patients whose GFR was compromised to less than 60 ml/min, and one weekly cisplatin dose was held if GFR fell below 30 ml/min until improvement of the kidney function. Overall, the mean cumulative cisplatin dose was 255 mg in the control group patients, and 154 mg in the intervention group patients. The mean ideal cumulative cisplatin dose based on individual patients' body surface areas would have been 311 mg and 183 mg in the control and intervention groups, respectively.

Analysis of any acute grade 3 or higher toxicities showed a 44.8% rate in the intervention versus 30% in the control group (Pearson’s chi squared = 1.4, *p* value: 0.24) (Tables 2, 3). There was a significantly higher rate of acute grade ≥ 3 GI toxicity in the intervention group (27.6%) compared with the control group (6.7%) (*p* value 0.032). Other than one control patient who developed severe ileus and constipation, and one intervention patient who developed severe enterocolitis, all other patients with grade ≥ 3 GI toxicities had severe diarrhea.

**Table 2.** Treatment toxicities in the study groups based on CTCAE 5 grading

	Grade ≤ 2	Grade 3	Grade 4	Grade 5
Any	37 (62.7%)	15 (25.4%)	5 (8.5%)	2 (3.4%)
Control (*n* = 30)	21 (70%)	7 (23.3%)	1 (3.3%)	1 (3.3%)
Intervention (*n* = 29)	16 (55.2%)	8 (27.6%)	4 (13.8%)	1 (3.4%)
GI	49 (83.1%)	6 (10.2%)	2 (3.4%)	2 (3.4%)
Control (*n* = 30)	28 (93.3%)	1 (3.3%)	0	1 (3.3%)
Intervention (*n* = 29)	21 (72.4%)	5 (17.2%)	2 (6.9%)	1 (3.4%)
GU	56 (94.9%)	1 (1.7%)	2 (3.4%)	0
Control (*n* = 30)	28 (93.3%)	1 (3.3%)	1 (3.3%)	0
Intervention (*n* = 29)	28 (96.6%)	0	1 (3.4%)	0
Hematologic	47 (79.7%)	11 (18.6%)	1 (1.7%)	0
Control (*n* = 30)	23 (76.7%)	7 (23.3%)	0	0
Intervention (*n* = 29)	24 (82.8%)	4 (13.8%)	1 (3.4%)	0
Skin	58 (98.3%)	1 (1.7%)	0 (0%)	0
Control (*n* = 30)	30 (100%)	0	0	0
Intervention (*n* = 29)	28 (96.6%)	1 (3.4%)	0	0
AKI	59 (100%)	0	0	0
Control (*n* = 30)	30 (100%)	0	0	0
Intervention (*n* = 29)	29 (100%)	0	0	0

*AKI*, acute kidney injury; *GI*, gastrointestinal; *GU*, genitourinary

**Table 3:** Comparison of grade 3 and higher toxicities between study groups

	Intervention	Control	*χ*^2^/*p* value
Grade ≥ 3 Any	13/29 (44.8%)	9/30 (30%)	1.4/0.24
Grade ≥ 3 GI	8/29 (27.6%)	2/30 (6.7%)	4.6/0.032
Grade ≥ 3 GU	1/29 (3.4%)	2/30 (6.7%)	0.32/0.57
Grade ≥ 3 Hematologic	5/29 (17.2%)	7/30 (23.3%)	0.34/0.56
Grade ≥ 3 Skin	1/29 (3.4%)	0/30 (0%)	1.05/0.30
Grade ≥ 3 AKI	0/29 (0%)	0/30 (0%)	-

*GI*, gastrointestinal, *GU*, genitourinary, *AKI*, acute kidney injury

The bowel volume receiving 45 Gy or higher (V45) was 388 cc [IQR 275–609] in the control group and 381 cc [IQR: 283.5–489] in the intervention group (Mann–Whitney *U*; *Z* = − 0.55, *p* value 0.58).

### Patterns of failure and follow-up

Ten patients in each group did not achieve a CCR. Of these, 6 intervention patients, and 5 control patients had local recurrences, 1 intervention patient, and 2 control patients had nodal recurrences, and 2 intervention patients and one control patient had distant metastases 3 months after the last IVRT session. One patient from the intervention group died because of severe diarrhea leading to hypokalemia and fatal arrhythmia, and one control patient developed severe ascites and peritonitis leading to septicemia and multi-organ failure. In addition, one control patient died because of acute myocardial infarction in the follow-up period before response evaluation. Patients with a locoregional recurrence were referred to a multidisciplinary tumor board for decision on whether to perform surgery or receive systemic therapy and patients with distant failure started palliative chemotherapy. In the follow-up evaluation with clinical examination and an abdominopelvic MRI 6 months after the last IVRT session, all complete responders were still disease-free with no evidence of locoregional recurrence.

## Discussion

Hypofractionated RT schedules benefit radiobiologically from a shorter treatment duration that would diminish tumoral repopulation. Using the corrected biological equivalent dose (BED) formula (Fowler 2010) which takes into consideration the tumoral repopulation after 21 days of RT initiation for cervical cancer tumors (Mahmoud et al. 2017), the calculated BED of external beam RT using an alpha/beta ratio of 10 for our experimental group was ~ 51 Gy and for the control group ~ 49 Gy. Also after adding the IVRT dose (28 Gy/4fr), the HR-CTV cumulative BED reached approximately 99 Gy and 97 Gy for the experimental and control groups, respectively. This shows that the experimental group RT schedule (40 Gy/15fr) was not inferiorly dosed, and benefited from a modestly higher corrected BED compared with the control group (45 Gy/25fr). These calculations and the expectation of higher compliance and more successful treatment conclusion in the intervention group patients was the underlying reason for our prediction of a higher CCR in this group. Despite a very small absolute difference in the 3-month CCR rate of 1.1% between the two groups, our interim analysis failed to show non-inferiority of the hypofractionated to conventional chemoradiation in the uterine cervix cancer patients. This is not an unexpected finding, because a sample size of 114 patients is required to establish a 90% power for this study and currently with the interim analysis, the upper boundary of the 95% confidence interval for the absolute difference (0.25) crosses the margin of non-inferiority. The Data Safety Monitoring Board performed a futility assessment with conditional power analysis after 50% patient accrual and was convinced that continuation of the trial until recruitment of the initially determined sample size would be necessary to statistically reject the null hypothesis of the experimental group inferiority. To be more precise, if the trial continuation leads to a similar CCR in the study groups as in this interim analysis, the observed absolute difference would have an upper 95% confidence interval boundary of less than the predetermined 15% margin which could lead to the rejection of null hypothesis and determination of non-inferiority.

In an unplanned subgroup analysis, we observed a significant difference in CCR rates seen with tumor sizes of less than 5 cm in favor of the intervention group and vice versa for tumor sizes of over 5 cm in favor of the control group. We can hypothesize that the explanation for this difference might be a better shrinkage of tumor within the 5-week RT duration in the control group compared with the shorter 3-week time for tumor shrinkage in the intervention group which might have resulted in a technically more complicated brachytherapy application and probably less favorable dose coverage of the tumor bulk periphery. On the other hand, the better CCR rate seen in the smaller tumors in the intervention group might be due to the beneficial effects of a 15-day-shorter OTT in the intervention group. According to Mazeron et al., OTT of more than 55 days is an independent prognostic factor for local control in cervix cancer. In addition, Gasinska et al. reported that OTT over 60 days is prognostic of overall survival, disease-free survival, and local control in cervix cancer. The reason underlying a longer OTT in our control and intervention groups (75 days ± SD 11 and 60 days ± SD 14, respectively) than recommended by these studies, was our strict decision to maintain a 7-day interval between brachytherapy fractions per the study protocol to increase the normal tissue repair and decrease the probability of severe toxicity.

Another advantage of a shorter treatment duration was seen as the intervention group patients received the full course of concurrent chemotherapy more frequently than the control group patients which trended toward significance (79.3% versus 60%, *p* value 0.11).

There are some points and limitations in this study that must be addressed. First, more than one-half of the study population were in FIGO stage IIIC1 which indicates that most of the patients presented to us in advanced stages. This situation was caused probably by the COVID-19 pandemic when patients sought medical advice very late in their disease course. This issue might affect the generalizability of our findings, especially to the patients in lower stages. Second, blinding the physicians in charge of the treatment delivery and the patients themselves was not possible due to the nature of RT treatment and the need for continuous monitoring of toxicities during treatment. This could potentially have introduced performance bias when planning for brachytherapy and treating the toxicities.

A more concerning limitation of this study is the fact that patients did not receive intensity-modulated radiation therapy (IMRT), and grade ≥ 3 GI toxicity was observed more frequently in the intervention group. This is in line with the fact that the bowel V45Gy was about twice the recommended volume (195 cc) by QUANTEC (Kavanagh et al. 2010) in both study groups and the higher dose per fraction in the intervention group might have contributed to the higher rates of acute grade ≥ 3 GI toxicity. Evidence for lower toxicity of the more conformal treatment modalities comes from multiple studies including the phase 1/2 SPARTACUS non-randomized trial, which used 30 Gy in 5 fractions with stereotactic body radiation therapy (SBRT) in 61 endometrial cancer patients and showed that this regimen was well tolerated by the patients with very low rates of grade 3 or higher GI/GU toxicity (< 2%) (Leung et al. 2022). Specifically, the NRG Oncology/RTOG 1203 compared IMRT and 3D conformal radiotherapy techniques in endometrial and cervix cancer patients regarding patient-reported GI and urinary toxicity, as well as the quality of life, and indicated improved measures in all outcomes with IMRT (Klopp et al. 2018). We presume that if IMRT was used instead of 3D conformal RT in our study, the bowel space as an organ at risk per QUANTEC recommendations would have been spared more readily, and the observed GI toxicities would have been significantly lower. Finally, the primary and secondary outcomes of this study were set for a 3-month interval from the last IVRT session, and despite performing a second follow-up 6 months after the last IVRT session confirming the disease-free status of all complete responders, a longer follow-up is needed to ascertain the safety of hypofractionation in both long-lasting oncologic efficacy and late toxicity endpoints.

To our knowledge, this is the first randomized clinical trial assessing the efficacy and toxicity of hypofractionated chemoradiation in uterine cervix cancer patients. Currently, two other trials from Mexico (NCT03750539) and Canada (NCT04583254) are recruiting with similar trial designs. Considering the above-mentioned issues, we will continue the second half of this trial using the IMRT technique and restrict our patients to those with a maximum tumor size of smaller than 5 cm.

## Conclusions

Despite a very small absolute difference in the 3-month CCR rate of 1.1%, our interim analysis failed to show non-inferiority of the hypofractionated chemoradiation to conventional chemoradiation in uterine cervix cancer patients. Considering the higher GI toxicity in the intervention group and the differential results in favor of hypofractionated chemoradiation in smaller cervix tumors, we will continue the second half of this trial using the IMRT technique and will restrict our patients to those with maximum tumor size of smaller than 5 cm.

## Data Availability

Research data are stored in an institutional repository and will be shared upon request to the corresponding author.
